# Impaired cerebral interstitial fluid dynamics in cerebral autosomal dominant arteriopathy with subcortical infarcts and leucoencephalopathy

**DOI:** 10.1093/braincomms/fcad349

**Published:** 2023-12-19

**Authors:** Shao-Lun Hsu, Yi-Chu Liao, Chia-Hung Wu, Feng-Chi Chang, Yung-Lin Chen, Kuan-Lin Lai, Chih-Ping Chung, Shih-Pin Chen, Yi-Chung Lee

**Affiliations:** Department of Neurology, Taipei Veterans General Hospital, Taipei 11217, Taiwan; Institute of Clinical Medicine, National Yang Ming Chiao Tung University, Taipei 11221, Taiwan; School of Medicine, National Yang Ming Chiao Tung University, Taipei 11221, Taiwan; Department of Neurology, National Yang Ming Chiao Tung University School of Medicine, Taipei 11221, Taiwan; Department of Neurology, Taipei Veterans General Hospital, Taipei 11217, Taiwan; School of Medicine, National Yang Ming Chiao Tung University, Taipei 11221, Taiwan; Department of Neurology, National Yang Ming Chiao Tung University School of Medicine, Taipei 11221, Taiwan; Brain Research Center, National Yang Ming Chiao Tung University, Taipei 11221, Taiwan; School of Medicine, National Yang Ming Chiao Tung University, Taipei 11221, Taiwan; Department of Radiology, Taipei Veterans General Hospital, Taipei 11217, Taiwan; School of Medicine, National Yang Ming Chiao Tung University, Taipei 11221, Taiwan; Department of Radiology, Taipei Veterans General Hospital, Taipei 11217, Taiwan; Institute of Biophotonics, National Yang Ming Chiao Tung University, Taipei 11221, Taiwan; Department of Neurology, Taipei Veterans General Hospital, Taipei 11217, Taiwan; School of Medicine, National Yang Ming Chiao Tung University, Taipei 11221, Taiwan; Department of Neurology, National Yang Ming Chiao Tung University School of Medicine, Taipei 11221, Taiwan; Brain Research Center, National Yang Ming Chiao Tung University, Taipei 11221, Taiwan; Department of Neurology, Taipei Veterans General Hospital, Taipei 11217, Taiwan; School of Medicine, National Yang Ming Chiao Tung University, Taipei 11221, Taiwan; Department of Neurology, National Yang Ming Chiao Tung University School of Medicine, Taipei 11221, Taiwan; Brain Research Center, National Yang Ming Chiao Tung University, Taipei 11221, Taiwan; Department of Neurology, Taipei Veterans General Hospital, Taipei 11217, Taiwan; Institute of Clinical Medicine, National Yang Ming Chiao Tung University, Taipei 11221, Taiwan; School of Medicine, National Yang Ming Chiao Tung University, Taipei 11221, Taiwan; Department of Neurology, National Yang Ming Chiao Tung University School of Medicine, Taipei 11221, Taiwan; Brain Research Center, National Yang Ming Chiao Tung University, Taipei 11221, Taiwan; Division of Translational Research, Department of Medical, Neurological Institute, Taipei Veterans General Hospital, Taipei 11217, Taiwan; Department of Neurology, Taipei Veterans General Hospital, Taipei 11217, Taiwan; School of Medicine, National Yang Ming Chiao Tung University, Taipei 11221, Taiwan; Department of Neurology, National Yang Ming Chiao Tung University School of Medicine, Taipei 11221, Taiwan; Brain Research Center, National Yang Ming Chiao Tung University, Taipei 11221, Taiwan; Center for Intelligent Drug Systems and Smart Bio-devices (IDS2B), National Yang Ming Chiao Tung University, Hsinchu 30010, Taiwan

**Keywords:** CADASIL, *NOTCH3* gene, glymphatic system, interstitial fluid dynamics, cerebral small vessel disease

## Abstract

Cerebral autosomal dominant arteriopathy with subcortical infarcts and leucoencephalopathy, caused by cysteine-altering variants in *NOTCH3*, is the most prevalent inherited cerebral small vessel disease. Impaired cerebral interstitial fluid dynamics has been proposed as one of the potential culprits of neurodegeneration and may play a critical role in the initiation and progression of cerebral small vessel disease. In the present study, we aimed to explore the cerebral interstitial fluid dynamics in patients with cerebral autosomal dominant arteriopathy with subcortical infarcts and leucoencephalopathy and to evaluate its association with clinical features, imaging biomarkers and disease severity of cerebral autosomal dominant arteriopathy with subcortical infarcts and leucoencephalopathy. Eighty-one participants carrying a cysteine-altering variant in *NOTCH3*, including 44 symptomatic cerebral autosomal dominant arteriopathy with subcortical infarcts and leucoencephalopathy patients and 37 preclinical carriers, and 21 age- and sex-matched healthy control individuals were recruited. All participants underwent brain MRI studies and neuropsychological evaluations. Cerebral interstitial fluid dynamics was investigated by using the non-invasive diffusion tensor image analysis along the perivascular space method. We found that cerebral autosomal dominant arteriopathy with subcortical infarcts and leucoencephalopathy patients exhibited significantly lower values of diffusion tensor image analysis along the perivascular space index comparing to preclinical carriers and healthy controls. For the 81 subjects carrying *NOTCH3* variants, older age and presence of hypertension were independently associated with decreased diffusion tensor image analysis along the perivascular space index. The degree of cerebral interstitial fluid dynamics was strongly related to the severity of cerebral small vessel disease imaging markers, with a positive correlation between diffusion tensor image analysis along the perivascular space index and brain parenchymal fraction and negative correlations between diffusion tensor image analysis along the perivascular space index and total volume of white matter hyperintensity, peak width of skeletonized mean diffusivity, lacune numbers and cerebral microbleed counts. In addition, diffusion tensor image analysis along the perivascular space index was a significant risk factor associated with the development of clinical symptoms of stroke or cognitive dysfunction in individuals carrying *NOTCH3* variants. In cerebral autosomal dominant arteriopathy with subcortical infarcts and leucoencephalopathy patients, diffusion tensor image analysis along the perivascular space index was significantly associated with Mini-Mental State Examination scores. Mediation analysis showed that compromised cerebral interstitial fluid dynamics was not only directly associated with cognitive dysfunction but also had an indirect effect on cognition by influencing brain atrophy, white matter disruption, lacunar lesions and cerebral microbleeds. In conclusion, cerebral interstitial fluid dynamics is impaired in cerebral autosomal dominant arteriopathy with subcortical infarcts and leucoencephalopathy and its disruption may play an important role in the pathogenesis of cerebral autosomal dominant arteriopathy with subcortical infarcts and leucoencephalopathy. Diffusion tensor image analysis along the perivascular space index may serve as a biomarker of disease severity for cerebral autosomal dominant arteriopathy with subcortical infarcts and leucoencephalopathy.

## Teaser

Hsu *et al*. utilized diffusion tensor imaging along perivascular space method and found that cerebral interstitial fluid dynamics in cerebral autosomal dominant arteriopathy with subcortical infarcts and leucoencephalopathy (CADASIL) patients is associated with their clinical features, imaging biomarkers and disease severity. These findings suggest that impaired cerebral interstitial fluid dynamics play a crucial role in the pathogenesis of CADASIL.

## Graphical Abstract

**Graphical Abstract:**
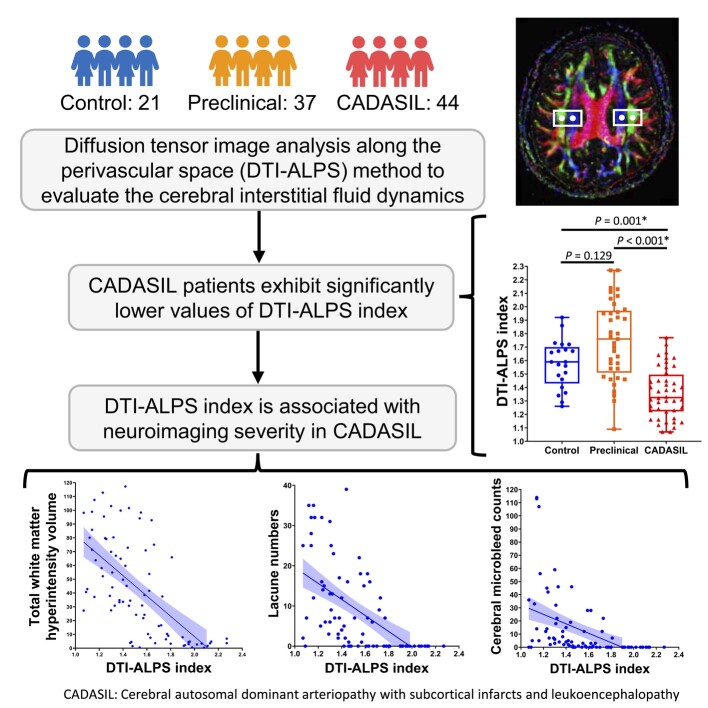

## Introduction

Cerebral autosomal dominant arteriopathy with subcortical infarcts and leucoencephalopathy (CADASIL) is the most common inherited cerebral small vessel disease (CSVD) worldwide.^1,2^ It is caused by cysteine-altering variants in the *NOTCH3* gene and is characterized by recurrent ischaemic stroke and vascular cognitive impairment at early or middle adulthood.^3,4^ The *NOTCH3* gene encodes a transmembrane receptor protein.^5^ In patients with cysteine-altering variants in *NOTCH3*, abnormal accumulation of the extracellular domain of mutant NOTCH3 protein around the vascular smooth muscle cells (VSMC) and pericytes of small arteries and arterioles would lead to the pathological hallmark of granular osmiophilic material (GOM) deposition seen on electron microscopy.^6,7^ However, GOM cannot explain all the vascular pathogenesis and neurodegeneration occurring in CADASIL patients along the disease course.^8^

Cerebral interstitial fluid (ISF) surrounds the parenchymal cell and plays a crucial role in maintaining the homeostasis of the brain by providing nutrients, removing waste products and facilitating communication between brain cells.^9^ The glymphatic system hypothesis proposed a brain-wide pathway that drives the cerebrospinal fluid (CSF) flow into the brain parenchyma via the periarterial spaces, followed by the transport of metabolic products or the elimination of metabolic waste through the convective ISF flux. The CSF-ISF mixture and brain metabolites are finally drained towards the perivenous spaces and cleared out of the brain.^10-12^ Additionally, the intramural periarterial drainage (IPAD) pathway acts as another ISF drainage system in charge of the elimination of metabolites from ISF along the basement membranes of VSMC in arterial wall towards the cervical lymph nodes.^13-15^ Impairment of cerebral ISF dynamics would change the neuronal environment, and has been implicated in the pathogenesis of neurodegenerative diseases like Alzheimer’s disease, as well as CSVD.^16-19^ In CADASIL, the accumulation of GOM deposition in the VSMC layer reduces the vascular tone of arterioles and compromises the cerebrovascular reactivity. This disruption results in a decline in periarterial CSF influx and altered cerebral ISF dynamics.^1,20-24^ Besides, the deposition of GOM may physically obstruct the IPAD and decrease the clearance of metabolic waste from ISF.^25-27^

Diffusion tensor image analysis along the perivascular space (DTI-ALPS) was recently introduced as a non-invasive method to calculate the ratio of the diffusivity of water in the direction of perivascular space and in the direction perpendicular to both major fibre tract and perivascular space at the level of the lateral ventricle body and which directly reflects the dynamics of the cerebral ISF.^28-30^ It has been shown that the DTI-ALPS index in patients with CSVD was associated with several imaging markers of CSVD, including the volume of white matter hyperintensity (WMH), cerebral microbleed (CMB) counts and the numbers of lacune, as well as correlated with the cognitive function.^31-34^

In the present study, we hypothesized that impairment of cerebral ISF dynamics might participate in the pathophysiology of CADASIL and could be associated with disease status and clinical severity in subjects with *NOTCH3* variants. We first compared the DTI-ALPS index between CADASIL patients and healthy individuals and analysed its difference between symptomatic CADASIL patients and preclinical *NOTCH3* cysteine-altering variant carriers. Then, we investigated which vascular risk factors or neuroimaging features were associated with a reduction in the DTI-ALPS index in the subjects with *NOTCH3* variants. Finally, we investigated whether impaired cerebral ISF dynamics is associated with the severity of cognitive dysfunction and disability in CADASIL patients.

## Materials and methods

### Subjects

A consecutive series of 81 participants who carried the cysteine-altering variants in *NOTCH3* were recruited from Department of Neurology, Taipei Veterans General Hospital between January 2019 and March 2023. The distribution of *NOTCH3* variants included S118C (2.5%, 2/81), R133C (3.7%, 3/81), C222S (1.2%, 1/81), R544C (homozygous: 1.2%, 1/81; heterozygous: 82.7%, 67/81), R545C (2.5%, 2/81), R558C (1.2%, 1/81), C977S (1.2%, 1/81), Y1069C (1.2%, 1/81) and C1250R (2.5%, 2/81). They were divided into two groups according to their clinical manifestations, including 37 preclinical carriers who never had neurological disorders except for migraine, and 44 symptomatic CADASIL patients who have developed ischaemic stroke, intracranial haemorrhage or cognitive dysfunction. We additionally enrolled 21 healthy individuals who were age- and sex-matched to the 81 subjects with *NOTCH3* variants and all of whom did not carry cysteine-altering variants in *NOTCH3* after the genetic analyses of exons 2 to 24 of *NOTCH3*. The genetic analysis was performed according to the previously published method.^35^ All study participants, including the subjects with *NOTCH3* variants and the healthy controls, underwent a brain MRI study using a 3-tesla MRI scanner (Signa, GE Healthcare, Milwaukee, WI). This study was approved by the institutional review board of Taipei Veterans General Hospital (TVGH IRB No. 2017-02-008A) and the written informed consents were obtained from all study participants.

### Clinical evaluation

A questionnaire was used to collect data from patients or/and their families about their demographic information, years of education, smoking habits, alcohol consumption and medical histories, including stroke (ischaemic stroke or intracerebral haemorrhage), cognitive impairment, hypertension, diabetes and hyperlipidaemia. For symptomatic CADASIL patients, the onset age of stroke or/and cognitive impairment was determined by medical records or questionnaire-based interviews by experienced neurologists. Disease duration was defined as the interval between the year of initial presentation (stroke or/and cognitive impairment) and the year of brain MRI examination. For the 81 subjects with *NOTCH3* variants, global cognitive performance was assessed by Mini-Mental State Examination (MMSE)^36^ and disability or dependence in the daily activities was measured by the modified Rankin Scale (mRS).^37^

### Brain MRI acquisition and analysis

DTI was performed with a *b*-value of 1000 s/mm^2^ (repetition time = 9500 milliseconds (ms); echo time = 81.5 ms; diffusion gradient encoding in 64 directions). High-resolution and 3D imaging including T2-fluid-attenuated inversion recovery (FLAIR) (1 mm slice thickness; repetition time = 6000 ms; echo time = 118 ms; inversion time = 1870 ms) was performed to evaluate WMH, and T_1_-weighted imaging (T1WI) (1 mm section thickness; repetition time = 7.1 ms; echo time = 2.7 ms; inversion time = 450 ms) was used to evaluate lacunar lesions. Susceptibility-weighted imaging (SWI) (2 mm section thickness; repetition time = 42.3 ms; echo time = 25.4 ms) was performed to detect CMBs. CMBs were defined as small, rounded hypointense areas that typically had a diameter of 2 to 5 mm but may have had a diameter up to 10 mm on SWI.^38^ Lacunar lesions were defined as parenchymal defects with a signal intensity corresponding to that of CSF on the T1WI with a diameter between 3 and 15 mm.^39^

**Figure 1:**
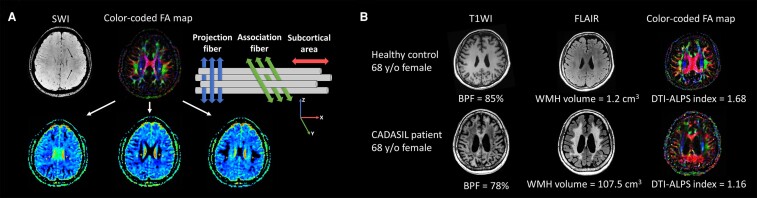
**Schematic drawing of the diffusivity measurement using the diffusion tensor image analysis along the perivascular space (DTI-ALPS) method and examples for calculating the image parameters.** (**A**) [*Top-left*] On axial susceptibility-weighted image at the level of the lateral ventricle body, the transmedullary vessels run along the *x*-axis (arrows). [*Top-middle*] Colour fractional anisotropy (FA) map of diffusion tensor image (DTI) demonstrates the distribution of projection neural fibres (*z*-axis: blue colour), association neural fibres (*y*-axis: green colour) and subcortical neural fibres (*x*-axis: red colour). [*Top-right*] The direction of the perivascular space (horizontal cylinders) is perpendicular to both projection and association neural fibres. The DTI-ALPS index was measured by quotient of mean of the *x*-projection and *x*-association areas over mean of the *y*-projection and *z*-association areas. [*Bottom*] Four fixed spherical regions of interest (ROIs) with 2 mm in diameter along the horizontal course of the transmedullary vessels were allocated in the areas of projection neural fibres (projection area) and association neural fibres (association area) on the colour-coded FA map bilaterally, then copied and pasted onto the three diffusivity maps to measure diffusivities along the *x*-, *y*- and *z*-axes (*left*, diffusivity map along the *x*-axis; *middle*, diffusivity map along *y*-axis; *right*, diffusivity map along *z*-axis). (**B**) The T_1_-weighted imaging (T1WI), T2-fluid-attenuated inversion recovery (FLAIR) and colour FA maps (from left to right) of a representative healthy control and a CADASIL patient were used to illustrate the calculation of brain parenchymal fraction (BPF), white matter hyperintensity (WMH) volume and DTI-ALPS index, respectively.

The DTI data were analysed by the VolumeViewer (version 11.0, GE Healthcare, Chicago, IL) with automatic synchronization and motion correction. The calculations of the diffusivities along *x*-, *y*- and *z*-directions were performed on the DTI-Advanced toolbox on VolumeViewer. The axial slice was selected at the level of the lateral ventricular body referencing on SWI, where the transmedullary vessels pass perpendicular to the ventricle. Fixed spherical regions of interest (ROIs) with 2 mm in diameter were placed onto the areas of the projection and association fibres that were differentiated based on the colour-mapped anisotropy maps generated automatically by the software (Fig. 1A). To minimize the influence of brain lesions and blood flow in the vessels, we ensured that the ROIs did not overlap with WMH, lacunar lesions, CMBs and visible vessels. This was accomplished by referencing the corresponding levels on SWI and T2-FLAIR images. The DTI-ALPS index that denotes the quotient of mean of the *x*-projection and *x*-association areas over mean of the *y*-projection and *z*-association areas was then calculated by the acquired values.^28^ The DTI-ALPS index was calculated on bilateral hemispheres separately and the values on both sides were averaged.

To quantify the severity of brain atrophy and WMH volume, we further performed volumetric analysis using the 3D slicer software (version 5.2.2).^40^ Measurement of intracranial cavity volume and brain parenchyma volume was conducted using T1WI after skull stripping. Brain parenchymal fraction (BPF) was assessed using the volume percentage of brain parenchyma (brain parenchyma volume/intracranial cavity volume × 100%).^41^ WMH volumes at the subcortical/deep brain regions and periventricular regions were segmented and calculated using T2-FLAIR images (Fig. 1B).^42^ MRI parameters of all study participants were evaluated by a single rater (S.-L.H.). Internal consistency of all image parameters was good with Cronbach’s α ranging from 0.84 to 0.99.^43^ We also performed inter-rater correlation analysis by comparing all image parameters of 10 randomly selected MRIs between our rater (S.-L.H.) and an experienced neuroradiology specialist (C.-H.W.), and the intraclass correlation coefficients ranged from 0.77 to 0.99.

Peak width of skeletonized mean diffusivity (PSMD) is an established and robust imaging marker for CSVD and is associated with microstructural disruption of white matter over the whole brain.^44^ The PSMD measure refers to the difference between the 5th and 95th percentiles of the voxel-based mean diffusivity values within the white matter tract skeleton that is calculated by using a fully automated shell script (http://www.psmd-marker.com/) on the preprocessed diffusion weighted imaging sequences.

### Statistical analyses

Analyses were performed with SPSS software (version 22.0; IBM). A two-tailed *P*-value of <0.05 was considered statistically significant. All data were presented as mean ± standard deviation (SD) (range) or number (percentage). Continuous variables were compared using *t*-test or Mann–Whitney U-test, and categorical variables were compared by chi-square test or Fisher’s exact test. Analysis of covariance with adjustment of age and sex was used to compare the imaging markers among healthy controls, preclinical carriers and symptomatic CADASIL patients.

To investigate which factors were determinants of the DTI-ALPS index, we used univariate regression analysis to identify clinical features or vascular risk factors that were significantly related to the DTI-ALPS index in all subjects carrying the *NOTCH3* variants, as well as subgroup analysis with preclinical carriers and CADASIL patients analysed separately. We also evaluated the associations between the DTI-ALPS index and CSVD imaging markers with generalized linear model adjusting for age, sex and hypertension.

Logistic regression was used to determine factors related to the presence of clinical symptoms in subjects carrying the *NOTCH3* variants and to estimate the corresponding odds ratio (OR) and 95% confidence interval (CI). The diagnostic accuracy of the factors significantly related to symptom onset was measured by receiver operating characteristic (ROC) curves with area under curve (AUC) method.

Mediation analysis was performed through the PROCESS macro for SPSS (model 4) using the bootstrap method with age, sex and education as covariates at a level of 95% confidence with 5000 bootstrap samples. The differences were considered statistically significant if the upper and lower 95% CI did not cross 0. The per cent of mediation (Pm) was calculated by indirect effect divided by total effect in order to study the weight of mediators in the total effect.

## Results

### Demographic and clinical features of the study participants

We enrolled 81 individuals with *NOTCH3* cysteine-altering variants (mean age = 57.2 ± 13.1 years, males/females = 36/45), including 37 preclinical carriers, and 44 symptomatic CADASIL patients who manifested with stroke or cognitive decline. In the symptomatic CADASIL patients, the average age at initial symptom onset was 59.6 ± 9.7 years. Thirty-seven of the 44 patients (84.1%) had experienced stroke and 36 patients (81.8%) had cognitive impairment. The average age of the 21 age- and sex-matched healthy controls was 57.1 ± 12.9 years, and 47.6% of them were males. The demographics, clinical manifestations and cardiovascular risk factors of the study participants are shown in Table 1.

**Table 1:** Demographic and clinical features of the subjects with *NOTCH3* variants and healthy controls

Variables	Healthy controls (*n* = 21)	All subjects with *NOTCH3* variants (*n* = 81)	*P*-value^a^	Preclinical carriers (*n* = 37)	CADASIL patients (*n* = 44)	*P*-value^a^
Males	10 (47.6)	36 (44.4)	0.797	10 (27)	24 (59.1)	0.004*
Age at exam, years	57.1 ± 12.9 (29–78)	57.2 ± 13.1 (27–80)	0.941	49.5 ± 13.1 (27–77)	63.6 ± 9.1 (41–80)	<0.001*
Education, years	16.3 ± 4.3 (6–22)	13.0 ± 4.3 (0–19)	0.002*	14.4 ± 3.1 (6–18)	11.9 ± 4.8 (0–19)	0.025*
MMSE score	29.5 ± 1.4 (24–30)	27.0 ± 5.2 (9–30)	0.001*	29.6 ± 0.6 (28–30)	24.7 ± 6.3 (9–30)	<0.001*
Modified Rankin’s scale	0.1 ± 0.3 (0–1)	0.99 ± 1.2 (0–4)	<0.001*	0.03 ± 0.2 (0–1)	1.8 ± 1.2 (0–4)	<0.001*
Clinical manifestations						
Stroke	-	37 (84.1)	-	-	37 (84.1)	-
Cognitive impairment	-	36 (81.8)	-	-	36 (81.8)	-
Age at onset, years	-	59.6 ± 9.7 (35–78)	-	-	59.6 ± 9.7 (35–78)	-
Disease duration, years	-	4.0 ± 3.6 (0–14)	-	-	4.0 ± 3.6 (0–14)	-
Cardiovascular risk factors						
Hypertension	6 (28.6)	30 (37)	0.469	5 (13.5)	25 (56.8)	<0.001*
Diabetes	0 (0)	8 (9.9)	0.201	2 (5.4)	6 (13.6)	0.279
Hyperlipidaemia	2 (9.5)	25 (30.9)	0.048*	8 (21.6)	17 (38.6)	0.099
Smoking	2 (9.5)	19 (23.5)	0.229	6 (16.2)	13 (29.5)	0.158
Alcohol consumption	0 (0)	19 (23.5)	0.011*	7 (18.9)	12 (27.3)	0.377

Values are presented as mean ± SD (range) or *n* (%).

CADASIL, cerebral autosomal dominant arteriopathy with subcortical infarcts and leucoencephalopathy; MMSE, Mini-Mental State Examination.

^a^ *P*-values were obtained from the *t*-test, Mann–Whitney U-test, chi-square test or Fisher’s exact test.

^*^ *P* < 0.05.

### Comparison of MRI parameters between subjects with *NOTCH3* variants and controls

**Figure 2:**
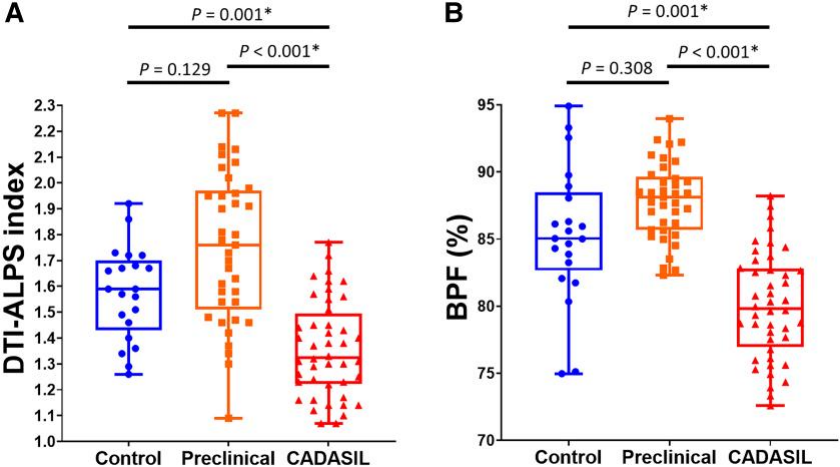
**Box and whisker plots for (A) DTI-ALPS index and (B) brain parenchymal fraction (BPF) in healthy controls, preclinical carriers and symptomatic CADASIL patients.** The box represents the interquartile range, with the horizontal line inside representing the median. The whiskers extend to the minimum and maximum values. *P*-value of <0.05 was considered statistically significant after adjusting for age and sex as confounders using analysis of covariance.

MRI parameters including DTI-ALPS index, BPF, WMH volume, PSMD, lacune numbers and CMB counts were compared among healthy controls, preclinical carriers and symptomatic CADASIL patients (Supplementary Table 1). After adjusting for age and sex effect, symptomatic CADASIL patients had significantly lower DTI-ALPS index, i.e. impaired cerebral ISF dynamics, in comparison to preclinical carriers or healthy controls (Fig. 2A). In addition, CADASIL patients had more profound brain atrophy (i.e. lower BPF) than preclinical carriers and healthy controls (Fig. 2B). As expected, CADASIL patients had significantly higher WMH volume, higher PSMD, more lacunes and greater CMB counts in comparison to controls or preclinical carriers (Supplementary Table 1). Although the average DTI-ALPS index and BPF seemed paradoxically increased in the preclinical carriers than controls, the difference was not statistically significant. Despite the *NOTCH3* variants carriers not displaying any clinical manifestations, their MRIs still had significantly larger WMH volume, more lacunes and CMBs and higher PSMD value in comparison to healthy controls (Supplementary Table 1).

### Factors related to decreased DTI-ALPS index in the subjects with *NOTCH3* variants

We then investigated which vascular risk factors were associated with a lower value of DTI-ALPS index in subjects carrying *NOTCH3* variants (Supplementary Table 2). Age at MRI examination (β = −0.016, *P* < 0.001) and presence of hypertension (β = −0.272, *P* < 0.001) were significantly associated with decreased DTI-ALPS index; whereas sex, diabetes, hyperlipidaemia, smoking and alcohol consumption were not related to the DTI-ALPS index. We further performed subgroup analysis by dividing the subjects into two groups according to the presence of clinical manifestations or not. We found that age and hypertension remained significantly associated with the DTI-ALPS index in the preclinical carriers, but they were no longer correlated with the reduced DTI-ALPS index in the CADASIL patients.

**Figure 3:**
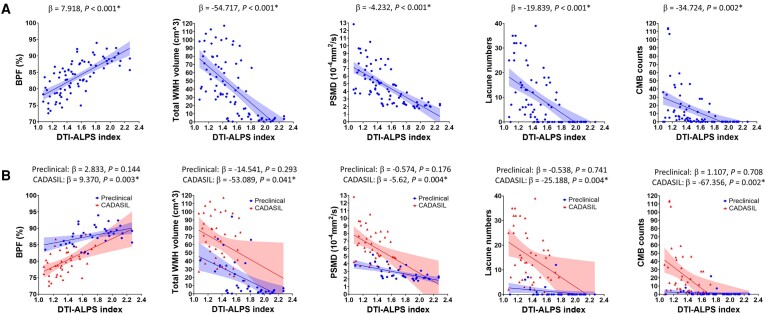
**Added variable plots illustrating the relationship between DTI-ALPS index and brain parenchymal fraction (BPF), total white matter hyperintensity (WMH) volume, peak width of skeletonized mean diffusivity (PSMD), lacune numbers and cerebral microbleed (CMB) counts in all subjects with *NOTCH3* variants (A) and preclinical carriers versus symptomatic CADASIL patients (B).** The plots show the disease stage-stratified relationship between DTI-ALPS index and other imaging markers. Shaded areas represent 95% confidence intervals. The beta coefficient and *P*-value of regression analysis are also shown in each plot. *P*-value of <0.05 was considered statistically significant.

We also investigated the associations between imaging markers of CSVD and DTI-ALPS index among the subjects with *NOTCH3* variants (Supplementary Table 3). After adjusting for age, sex and hypertension, the DTI-ALPS index showed a positive correlation with BPF (β = 7.918, *P* < 0.001) but negative correlations with total WMH volume (β = −54.717, *P* < 0.001), PSMD (β = −4.232, *P* < 0.001), lacune numbers (β = −19.839, *P* < 0.001) and CMB counts (β = −34.724, *P* = 0.002) (Fig. 3A). In the subgroup analysis, the correlations between DTI-ALPS index and imaging markers remained significant in the symptomatic CADASIL patients but not in the preclinical carriers (Fig. 3B).

### A reduced DTI-ALPS index could distinguish symptomatic CADASIL from preclinical carrier state

It is interesting to know which factors could determine the symptom onset for subjects with *NOTCH3* variants. In univariate logistic regression, older age (OR = 1.11), male gender (OR = 3.90), less education (OR = 0.86) and history of hypertension (OR = 8.42) were all risk factors associated with the development of clinical symptoms of stroke or cognitive dysfunction in individual carrying *NOTCH3* variants (Table 2). When including DTI-ALPS index into the multivariable regression model, we found that only male gender (OR = 5.47) and the DTI-ALPS index (OR = 0.009) were independent factors significantly associating with whether an individual with a *NOTCH3* variant would remain to be asymptomatic or not.

**Table 2:** The risk factors associated with the presence of clinical symptoms in the subjects with *NOTCH3* variants

Variable	Univariate regression analysis Odds ratio (95% CI), *P*-value	Multivariate regression analysis Odds ratio (95% CI), *P*-value	
		Model 1^a^	Model 2^b^
Age, years	1.114 (1.06, 1.171), <0.001*	1.098 (1.032, 1.167), 0.003*	1.048 (0.978, 1.123), 0.184
Sex (male)	3.900 (1.52, 10.005), 0.005*	6.143 (1.69, 22.322), 0.006*	5.465 (1.339, 22.31), 0.018*
Education, years	0.860 (0.761, 0.971), 0.015*	0.914 (0.777, 1.074), 0.275	0.930 (0.782, 1.107), 0.416
Hypertension	8.421 (2.76, 25.692), <0.001*	4.469 (1.205, 16.576), 0.025*	3.438 (0.880, 13.438), 0.076
Diabetes	2.763 (0.523, 14.603), 0.231	-	-
Hyperlipidaemia	2.282 (0.848, 6.145), 0.102	-	-
Smoking	2.167 (0.730, 6.431), 0.164	-	-
Alcohol consumption	1.607 (0.559, 4.624), 0.379	-	-
DTI-ALPS index	0.001 (8.7 × 10^−4^, 0.025), <0.001*	-	0.009 (2.67 × 10^−4^, 0.284), 0.008*

DTI-ALPS, diffusion tensor image analysis along the perivascular space; CI, confidence interval.

^a^Model 1: including age, sex, education and hypertension.

^b^Model 2: including age, sex, education, hypertension and DTI-ALPS index.

^*^ *P* < 0.05.

We then explored the capacity of the DTI-ALPS index to differentiate symptomatic CADASIL from preclinical *NOTCH3* carriers (Supplementary Fig. 1, Supplementary Table 4). The DTI-ALPS index had a good diagnostic accuracy to distinguish symptomatic CADASIL patients from preclinical carriers with an AUC of 0.87 (95% CI = 0.79 to 0.95), which was comparable to the diagnostic accuracy when combining the DTI-ALPS index with other vascular risk factors in the ROC analysis [AUC of ∼0.90 (95% CI = 0.83 to 0.97)].

### Regression analysis and mediation analysis for the correlations between DTI-ALPS index and disease severity in CADASL patients

We further investigated which imaging markers were significantly associated with the severity of cognitive dysfunction and disability (i.e. MMSE and mRS) in the CADASIL patients (Table 3). In univariate regression analysis, the DTI-ALPS index, BPF, WMH volume, PSMD, lacune numbers and CMB counts were all correlated with MMSE and mRS. In multivariate regression analysis, only the DTI-ALPS index (β = 10.928, *P* = 0.008) and CMB counts (β = −0.114, *P* < 0.001) were independently associated with MMSE, while PSMD (β = 0.161, *P* = 0.022) and CMB counts (β = 0.022, *P* < 0.001) were independently associated with the mRS.

**Table 3:** The correlation between imaging markers and disease severity in the CADASIL patients

Variables		Univariate regression analysis		Multivariate regression analysis^a^	
		MMSE	mRS	MMSE	mRS
		β (95% CI), *P*-value			
Age, years		−0.106 (−0.318, 0.106), 0.318	0.026 (−0.014, 0.065), 0.195		
Education, years		0.183 (−0.217, 0.583), 0.361	−0.027 (−0.102, 0.048), 0.465		
DTI-ALPS index		18.370 (9.599, 27.140), <0.001*	−3.016 (−4.733, −1.299), 0.001*	10.928 (2.968, 18.888), 0.008*	
Brain parenchymal fraction, %		0.773 (0.342, 1.204), 0.001*	−0.149 (−0.229, −0.069), <0.001*		
Total WMH volume		−0.096 (−0.156, −0.037), 0.002*	0.021 (0.011, 0.032), <0.001*		
PSMD, 10^−4^mm^2^/s		−1.670 (−2.368, −0.971), <0.001*	0.311 (0.180, 0.441), <0.001*		0.161 (0.024, 0.298), 0.022*
Lacune numbers		−0.294 (−0.455, −0.134), 0.001*	0.048 (0.017, 0.079), 0.003*		
Cerebral microbleed counts		−0.143 (−0.193 −0.093), <0.001*	0.027 (0.018, 0.036), <0.001*	−0.114 (−0.165, −0.064), <0.001*	0.022 (0.013, 0.032), <0.001*

CADASIL, cerebral autosomal dominant arteriopathy with subcortical infarcts and leucoencephalopathy; CI, confidence interval; DTI-ALPS, diffusion tensor image analysis along the perivascular space; MMSE, Mini-Mental Status Examination; mRS, modified Rankin Scale; PSMD, peak width of skeletonized mean diffusivity.

^a^Stepwise regression analysis.

^*^ *P* < 0.05.

**Figure 4:**
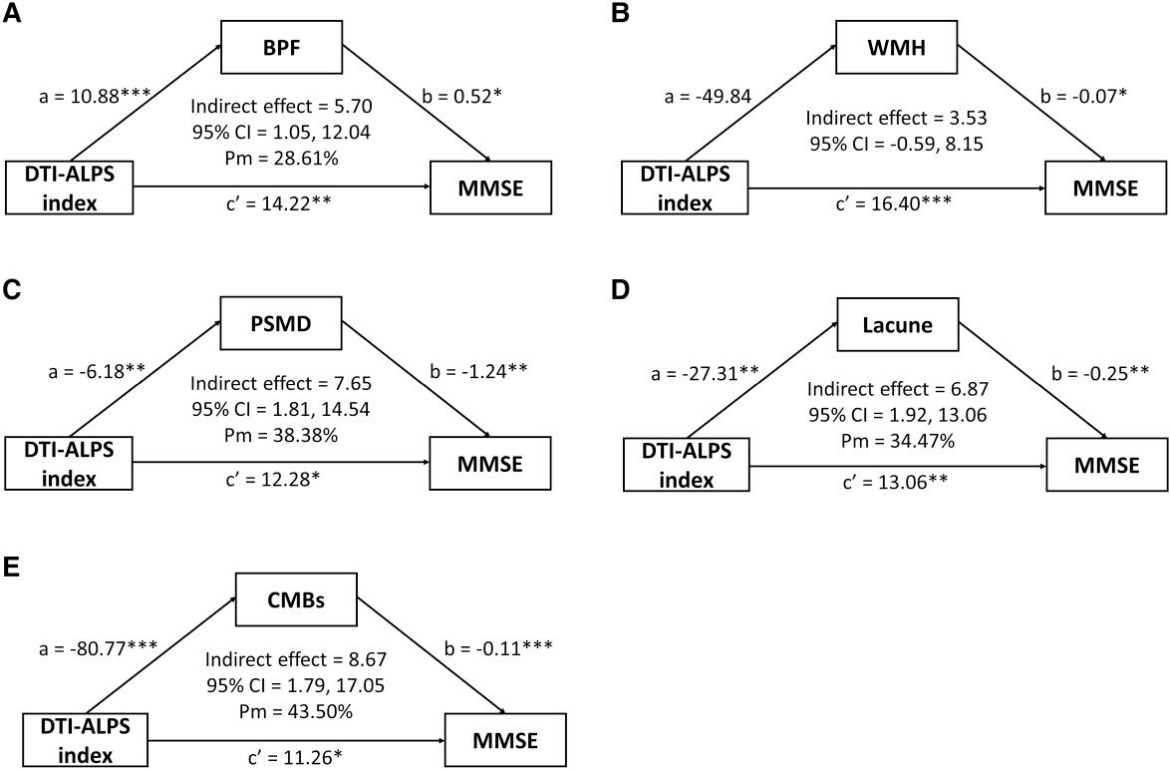
**Mediating effects among DTI-ALPS index, imaging markers and Mini-Mental Status Examination (MMSE) scores in CADASIL patients with age, sex and education level adjustment.** The DTI-ALPS index is the predictor, (**A**) brain parenchymal fraction (BPF), (**B**) total white matter hyperintensity (WMH) volume, (**C**) peak width of skeletonized mean diffusivity (PSMD), (**D**) lacune numbers and (**E**) cerebral microbleed (CMB) counts are the mediators, and the MMSE scores represent the disease severity. a and b indicate the indirect effect of the DTI-ALPS index on cognitive outcomes via the imaging markers and c′ indicates the direct effect of the DTI-ALPS index on MMSE score. Each data set was analysed by the bootstrapping method with 5000 resamples. CI, confidence interval; Pm, per cent mediation = Indirect effect/total effect × 100%, *P < 0.05, **P < 0.01; ***P < 0.001.

Finally, mediation analyses were used to detect the mediators in the relationship between DTI-ALPS index and cognition (MMSE). After adjusting for age, sex and education years, BPF (indirect effect = 5.70; 95% CI = 0.95 to 12.02; Pm = 28.61%), PSMD (indirect effect = 7.65; 95% CI = 1.81 to 14.54; Pm = 38.38%), lacune numbers (indirect effect = 6.87; 95% CI = 2.00 to 13.20; Pm = 34.47%) and CMB counts (indirect effect = 8.67; 95% CI = 1.80 to 16.90; Pm = 43.50%) were significant factors mediating the relationship between the DTI-ALPS index and MMSE (Fig. 4). Therefore, the decline of DTI-ALPS index is not only directly associated with cognitive dysfunction but also has an indirect effect on cognition through affecting brain atrophy, white matter disruption, lacunar lesions and CMBs.

## Discussion

In the present study, we investigated the role of impaired cerebral ISF dynamics in CADASIL using DTI-ALPS through analysing the clinical and brain MRI features of 81 subjects with *NOTCH3* variants, including 44 symptomatic CADASIL patients and 37 preclinical carriers, and another 21 healthy control individuals. There are four major findings, which suggest a strong link between impaired cerebral ISF dynamics and disease severity of CADASIL. First, symptomatic CADASIL patients had a significantly lower DTI-ALPS index compared to that of preclinical carriers and healthy controls. Second, there were significant correlations between DTI-ALPS index and multiple MRI markers of CSVD, including WMH volume, lacune numbers, CMB counts, BPF and PSMD, even after adjusting for age and hypertension. The findings supported the association between DTI-ALPS index and disease severity of CSVD in subjects with *NOTCH3* variants. Third, DTI-ALPS index can serve as a marker to distinguish between symptomatic CADASIL patients and preclinical carriers. Finally, impaired cerebral ISF dynamics is closely associated with cognitive decline in the CADASIL patients, and this effect is partially mediated through brain atrophy, white matter disruption, lacunar lesions and CMBs.

The association between DTI-ALPS index and disease severity of CADASIL suggests that impaired cerebral ISF dynamics is implicated in the pathophysiology of CADASIL. In the glymphatic hypothesis, the bulk flow of CSF in the perivascular space is driven by cerebral arterial pulsation initiated by the cardiac and respiratory cycles that promote the influx of CSF to the brain parenchyma, and facilitate the clearance of interstitial solutes and wastes.^10,24,45^ In CADASIL, the accumulation of GOM deposition in the VSMC layer could reduce the vascular tone of arterioles and disrupt the periarterial CSF influx pathway.^1,20,21^ Additionally, previous studies have demonstrated a reduced cerebrovascular reactivity in CADASIL patients. The compromised autoregulation of the cerebrovascular network and altered intracranial haemodynamics might result in a decreased CSF-ISF bulk flow in CADASIL patients.^22-24^ Another possible pathogenesis of CADASIL is that the deposition of GOM physically block the IPAD that eliminates the cerebral metabolites from the ISF along the basement membranes of VSMC in arterial wall towards the cervical lymph nodes.^8,15,25-27^ Besides, directly disrupt VSMC function by GOM deposition may cause failure of the vasomotion, the spontaneous low-frequency contraction and relaxation of VSMC, which is considered the primary driving force of IPAD.^14,45^ In the present study, we investigated the dynamics of cerebral ISF in CADASIL patients using DTI-ALPS and found significantly lower DTI-ALPS index in the symptomatic CADASIL patients than in healthy controls.

CADASIL appears to have a profound negative impact on the DTI-ALPS index. Previous studies showed that hypertension and aging compromise the dynamics of cerebral ISF.^21,46^ It has been noted that elevated blood pressure would increase backflow, thereby reduce the net flow in the perivascular space and cause decreased CSF influx.^21,24^ Similarly, age has been demonstrated as a crucial factor influencing the DTI-ALPS index value, typically peaking in the age of 30s or 40s and gradually declining with aging.^46,47^ Surprisingly, our study revealed that hypertension and age were associated with the DTI-ALPS index only in preclinical carriers with *NOTCH3* variants, but the relationship was less evident in symptomatic CADASIL patients. This finding suggests that the pathophysiological changes associated with symptomatic CADASIL may have a profound impact on the DTI-ALPS index that outweigh the influence of hypertension or age on the DTI-ALPS index.

Impaired cerebral ISF dynamics seems to be a common feature in patients with CSVD, especially for CADASIL. Several studies have shown that a lower DTI-ALPS index is associated with a greater burden of WMH and CMBs, lacune numbers, profound brain atrophy and more severe cognitive impairment in patients with CSVD.^31-34^ Cerebral amyloid angiopathy (CAA) is a subtype of CSVD, characterized by deposition of amyloid β in the vessel walls of penetrating and surface arteries.^48^ Recent studies have also demonstrated impaired cerebral ISF dynamics in patients with CAA. The values of DTI-ALPS index in CAA patients were significantly associated with CSVD imaging markers, cognitive impairment and disease recurrence.^49^ In our study, we found that DTI-ALPS index in CADASIL patients was significantly associated with BPF, total WMH volume, PSMD, lacune numbers and CMB counts, which implies that impaired cerebral ISF dynamics is associated to the progression of neuroimaging biomarkers in CADASIL.

DTI-ALPS index could potentially serve as an indicator for the conversion of preclinical status to clinical manifestations in individuals carrying *NOTCH3* variants. In individuals carrying *NOTCH3* variants in our cohort, older age, male gender and hypertension were independent risk factors for the conversion of preclinical carriers to symptomatic patients. Interestingly, in multivariable regression, we found that a lower DTI-ALPS index was an independent risk factor for *NOTCH3* variant carriers to become symptomatic (i.e. developing stroke or cognitive dysfunction) even after adjusting for age, sex, education years and hypertension. Moreover, by comparing the AUC in the ROC analysis, we found that the DTI-ALPS index had better diagnostic accuracy to differentiate symptomatic CADASIL patients from preclinical carriers than any other vascular risk factors. A recent study that enrolled 2219 community-dwelling Chinese participants also demonstrated that adding the DTI-ALPS index to conventional models based on vascular risk factors enhanced the predictive ability for CSVD.^33^ These findings support the idea that DTI-ALPS index can serve as an indicator to monitor the disease severity of CADASIL and a reduced DTI-ALPS index may suggest a further worsening of the disease.

Additional findings from the present study provide further support for the involvement of impaired cerebral ISF dynamics in the disease severity of CADASIL. Notably, significant associations were observed between DTI-ALPS index and both cognitive function (MMSE) and disability (mRS) in CADASIL patients. These associations closely resemble the findings reported in previous studies of CSVD or Alzheimer's disease.^28,31-33^ Furthermore, several imaging features of CSVD, including as the numbers of lacunes, burdens of WMHs, PSMD, CMBs and the degree of brain atrophy, have been previously associated with cognitive impairment in CADASIL patients.^44,50-53^ However, utilizing multivariable regression analysis to assess the relationship between cognitive function, the CSVD imaging markers, age, education and the DTI-ALPS index in CADASIL patients, we found that the DTI-ALPS index demonstrated an independent correlation with MMSE scores (β = 10.928, *P* = 0.008). These findings underscore the crucial role of the DTI-ALPS index in disease severity of CADASIL patients. In addition to its independent correlation, the DTI-ALPS index may also have indirect associations with cognitive function in CADASIL patients through other factors. Given that DTI-ALPS index exhibited significant associations with multiple CSVD imaging markers in CADASIL patients (Supplementary Table 3) and in other CSVD patient groups,^31-34^ we conducted a mediation analysis to elucidate the role of the CSVD imaging markers in the indirect association between DTI-ALPS index and MMSE scores. After adjusting for age, sex and education, our findings revealed that brain atrophy (lower BPF), white matter disruption (higher PSMD), lacunar lesions and CMBs emerge as significant mediators in the connection between the DTI-ALPS index and cognitive function.

In the present study, it remains unclear why the mean values of the DTI-ALPS index and BPF were relatively higher in preclinical carriers than in healthy controls, despite no significant difference being found after adjusting for sex and age. To assess the reliability of this phenomenon, larger sample sizes of preclinical carriers and age- and sex-matched healthy controls are required in future studies.

There are several limitations in this study. First, CADASIL is not a common disease, so the case number was modest in the present study. However, we enrolled subjects in different stages of disease and successfully elucidated the relationship between DTI-ALPS index, imaging markers and clinical severity along the disease course. Second, all patients in this study were of Han ethnicity, and the majority carried the *NOTCH3* p.R544C variant (84%, 68/81). Therefore, whether the results of this study can be extrapolated to Western populations remains to be confirmed by further studies. Thirdly, this is a cross-sectional study. Further longitudinal studies are needed to clearly confirm the predictive value of DTI-ALPS index as an indicator for impending disease progression of CADASIL.

In conclusion, our study clearly demonstrated impaired cerebral ISF dynamics in patients with CADASIL, whereas preclinical carriers of *NOTCH3* variants exhibited normal cerebral ISF dynamics comparable to that of healthy controls. Furthermore, we observed that impaired cerebral ISF dynamics correlates with disease severity in CADASIL. This relationship is further mediated by brain atrophy, white matter disruption, lacunar lesions and CMBs. Our findings strongly suggest that impaired cerebral ISF dynamics may participate in the pathogenesis of CADASIL, while the DTI-ALPS index can serve as a valuable marker for monitoring the disease progression of CADASIL.

## Supplementary Material

fcad349_Supplementary_Data

## Data Availability

The data that support the findings of this study are available from the corresponding authors. Data will be shared on reasonable request and after ethics approval if requested by other investigators.

## References

[1] Chabriat H, Joutel A, Dichgans M, Tournier-Lasserve E, Bousser MG. Cadasil. Lancet Neurol. 2009;8(7):643–653. 19539236 10.1016/S1474-4422(09)70127-9

[2] Choi JC . Genetics of cerebral small vessel disease. J Stroke. 2015;17(1):7–16. 25692103 10.5853/jos.2015.17.1.7 PMC4325630

[3] Joutel A, Corpechot C, Ducros A, et al Notch3 mutations in CADASIL, a hereditary adult-onset condition causing stroke and dementia. Nature. 1996;383(6602):707–710. 8878478 10.1038/383707a0

[4] Tikka S, Mykkanen K, Ruchoux MM, et al Congruence between NOTCH3 mutations and GOM in 131 CADASIL patients. Brain. 2009; 132(Pt 4):933–939. 19174371 10.1093/brain/awn364 PMC2668941

[5] Bellavia D, Checquolo S, Campese AF, Felli MP, Gulino A, Screpanti I. Notch3: From subtle structural differences to functional diversity. Oncogene. 2008;27(38):5092–5098. 18758477 10.1038/onc.2008.230

[6] Ishiko A, Shimizu A, Nagata E, Takahashi K, Tabira T, Suzuki N. Notch3 ectodomain is a major component of granular osmiophilic material (GOM) in CADASIL. Acta Neuropathol. 2006;112(3):333–339. 16871402 10.1007/s00401-006-0116-2

[7] Ruchoux MM, Chabriat H, Bousser MG, Baudrimont M, Tournier-Lasserve E. Presence of ultrastructural arterial lesions in muscle and skin vessels of patients with CADASIL. Stroke. 1994;25(11):2291–2292. 7974561 10.1161/01.str.25.11.2291

[8] Yamamoto Y, Liao YC, Lee YC, Ihara M, Choi JC. Update on the epidemiology, pathogenesis, and biomarkers of cerebral autosomal dominant arteriopathy with subcortical infarcts and leukoencephalopathy. J Clin Neurol. 2023;19(1):12–27. 36606642 10.3988/jcn.2023.19.1.12 PMC9833879

[9] Hladky SB, Barrand MA. Mechanisms of fluid movement into, through and out of the brain: Evaluation of the evidence. Fluids Barriers CNS. 2014;11(1):26. 25678956 10.1186/2045-8118-11-26 PMC4326185

[10] Iliff JJ, Wang M, Liao Y, et al A paravascular pathway facilitates CSF flow through the brain parenchyma and the clearance of interstitial solutes, including amyloid beta. Sci Transl Med. 2012;4(147):147ra111. 10.1126/scitranslmed.3003748 PMC3551275 22896675

[11] Iliff JJ, Lee H, Yu M, et al Brain-wide pathway for waste clearance captured by contrast-enhanced MRI. J Clin Invest. 2013;123(3):1299–1309. 23434588 10.1172/JCI67677 PMC3582150

[12] Rasmussen MK, Mestre H, Nedergaard M. Fluid transport in the brain. Physiol Rev. 2022;102(2):1025–1151. 33949874 10.1152/physrev.00031.2020 PMC8897154

[13] Carare RO, Bernardes-Silva M, Newman TA, et al Solutes, but not cells, drain from the brain parenchyma along basement membranes of capillaries and arteries: Significance for cerebral amyloid angiopathy and neuroimmunology. Neuropathol Appl Neurobiol. 2008;34(2):131–144. 18208483 10.1111/j.1365-2990.2007.00926.x

[14] Aldea R, Weller RO, Wilcock DM, Carare RO, Richardson G. Cerebrovascular smooth muscle cells as the drivers of intramural periarterial drainage of the brain. Front Aging Neurosci. 2019;11:1. 30740048 10.3389/fnagi.2019.00001 PMC6357927

[15] Carare RO, Aldea R, Agarwal N, et al Clearance of interstitial fluid (ISF) and CSF (CLIC) group—part of vascular Professional Interest Area (PIA): Cerebrovascular disease and the failure of elimination of amyloid-beta from the brain and retina with age and Alzheimer’s disease—opportunities for therapy. Alzheimers Dement (Amst). 2020; 12(1):e12053. 32775596 10.1002/dad2.12053 PMC7396859

[16] Bohr T, Hjorth PG, Holst SC, et al The glymphatic system: Current understanding and modeling. iScience. 2022;25(9):104987. 36093063 10.1016/j.isci.2022.104987 PMC9460186

[17] Verheggen ICM, Van Boxtel MPJ, Verhey FRJ, Jansen JFA, Backes WH. Interaction between blood–brain barrier and glymphatic system in solute clearance. Neurosci Biobehav Rev. 2018;90:26–33. 29608988 10.1016/j.neubiorev.2018.03.028

[18] Wardlaw JM, Smith C, Dichgans M. Small vessel disease: Mechanisms and clinical implications. Lancet Neurol. 2019;18(7):684–696. 31097385 10.1016/S1474-4422(19)30079-1

[19] Rasmussen MK, Mestre H, Nedergaard M. The glymphatic pathway in neurological disorders. Lancet Neurol. Nov 2018;17(11):1016–1024. 30353860 10.1016/S1474-4422(18)30318-1 PMC6261373

[20] Shi Y, Thrippleton MJ, Blair GW, et al Small vessel disease is associated with altered cerebrovascular pulsatility but not resting cerebral blood flow. J Cereb Blood Flow Metab. 2020;40(1):85–99. 30295558 10.1177/0271678X18803956 PMC6928551

[21] Mestre H, Tithof J, Du T, et al Flow of cerebrospinal fluid is driven by arterial pulsations and is reduced in hypertension. Nat Commun. 2018;9(1):4878. 30451853 10.1038/s41467-018-07318-3 PMC6242982

[22] Blair GW, Thrippleton MJ, Shi Y, et al Intracranial hemodynamic relationships in patients with cerebral small vessel disease. Neurology. 2020;94(21):e2258–e2269. 32366534 10.1212/WNL.0000000000009483 PMC7357294

[23] Pfefferkorn T, von Stuckrad-Barre S, Herzog J, Gasser T, Hamann GF, Dichgans M. Reduced cerebrovascular CO_2_ reactivity in CADASIL: A transcranial Doppler sonography study. Stroke. 2001;32(1):17–21. 11136908 10.1161/01.str.32.1.17

[24] Iliff JJ, Wang M, Zeppenfeld DM, et al Cerebral arterial pulsation drives paravascular CSF-interstitial fluid exchange in the murine brain. J Neurosci. 2013;33(46):18190–18199. 24227727 10.1523/JNEUROSCI.1592-13.2013 PMC3866416

[25] Takahashi K, Adachi K, Yoshizaki K, Kunimoto S, Kalaria RN, Watanabe A. Mutations in NOTCH3 cause the formation and retention of aggregates in the endoplasmic reticulum, leading to impaired cell proliferation. Hum Mol Genet. 2010;19(1):79–89. 19825845 10.1093/hmg/ddp468

[26] Hanemaaijer ES, Panahi M, Swaddiwudhipong N, et al Autophagy-lysosomal defect in human CADASIL vascular smooth muscle cells. Eur J Cell Biol. 2018;97(8):557–567. 30392756 10.1016/j.ejcb.2018.10.001

[27] Yamamoto Y, Craggs LJ, Watanabe A, et al Brain microvascular accumulation and distribution of the NOTCH3 ectodomain and granular osmiophilic material in CADASIL. J Neuropathol Exp Neurol. 2013;72(5):416–431. 23584202 10.1097/NEN.0b013e31829020b5

[28] Taoka T, Masutani Y, Kawai H, et al Evaluation of glymphatic system activity with the diffusion MR technique: Diffusion tensor image analysis along the perivascular space (DTI-ALPS) in Alzheimer’s disease cases. Jpn J Radiol. 2017;35(4):172–178. 28197821 10.1007/s11604-017-0617-z

[29] Taoka T, Ito R, Nakamichi R, Nakane T, Kawai H, Naganawa S. Interstitial fluidopathy of the central nervous system: An umbrella term for disorders with impaired neurofluid dynamics. Magn Reson Med Sci. 2022. doi:10.2463/mrms.rev.2022-0012. PMC10838724 36436975

[30] Taoka T, Ito R, Nakamichi R, et al Reproducibility of diffusion tensor image analysis along the perivascular space (DTI-ALPS) for evaluating interstitial fluid diffusivity and glymphatic function: CHanges in Alps index on Multiple conditiON acquIsition eXperiment (CHAMONIX) study. Jpn J Radiol. 2022;40(2):147–158. 34390452 10.1007/s11604-021-01187-5 PMC8803717

[31] Tang J, Zhang M, Liu N, et al The association between glymphatic system dysfunction and cognitive impairment in cerebral small vessel disease. Front Aging Neurosci. 2022;14:916633. 35813943 10.3389/fnagi.2022.916633 PMC9263395

[32] Zhang W, Zhou Y, Wang J, et al Glymphatic clearance function in patients with cerebral small vessel disease. Neuroimage. 2021;238:118257. 34118396 10.1016/j.neuroimage.2021.118257

[33] Tian Y, Cai X, Zhou Y, et al Impaired glymphatic system as evidenced by low diffusivity along perivascular spaces is associated with cerebral small vessel disease: A population-based study. Stroke Vasc Neurol. 2023;8(5):413–423. 37045543 10.1136/svn-2022-002191 PMC10647865

[34] Ke Z, Mo Y, Li J, et al Glymphatic dysfunction mediates the influence of white matter hyperintensities on episodic memory in cerebral small vessel disease. Brain Sci. 2022; 12(12):1611. 36552071 10.3390/brainsci12121611 PMC9775074

[35] Liao YC, Hsiao CT, Fuh JL, et al Characterization of CADASIL among the Han Chinese in Taiwan: Distinct genotypic and phenotypic profiles. PLoS One. 2015;10(8):e0136501. 26308724 10.1371/journal.pone.0136501 PMC4550240

[36] Folstein MF, Folstein SE, McHugh PR. “Mini-mental state”. A practical method for grading the cognitive state of patients for the clinician. J Psychiatr Res. 1975;12(3):189–198. 1202204 10.1016/0022-3956(75)90026-6

[37] Banks JL, Marotta CA. Outcomes validity and reliability of the modified Rankin scale: Implications for stroke clinical trials: A literature review and synthesis. Stroke. 2007;38(3):1091–1096. 17272767 10.1161/01.STR.0000258355.23810.c6

[38] Greenberg SM, Vernooij MW, Cordonnier C, et al Cerebral microbleeds: A guide to detection and interpretation. Lancet Neurol. 2009;8(2):165–174. 19161908 10.1016/S1474-4422(09)70013-4 PMC3414436

[39] Bokura H, Kobayashi S, Yamaguchi S. Distinguishing silent lacunar infarction from enlarged Virchow–Robin spaces: A magnetic resonance imaging and pathological study. J Neurol. 1998;245(2):116–122. 9507419 10.1007/s004150050189

[40] Fedorov A, Beichel R, Kalpathy-Cramer J, et al 3D slicer as an image computing platform for the Quantitative Imaging Network. Magn Reson Imaging. 2012;30(9):1323–1341. 22770690 10.1016/j.mri.2012.05.001 PMC3466397

[41] Rudick RA, Fisher E, Lee JC, Simon J, Jacobs L. Use of the brain parenchymal fraction to measure whole brain atrophy in relapsing-remitting MS. Multiple Sclerosis Collaborative Research Group. Neurology. 1999;53(8):1698–1704. 10563615 10.1212/wnl.53.8.1698

[42] DeCarli C, Fletcher E, Ramey V, Harvey D, Jagust WJ. Anatomical mapping of white matter hyperintensities (WMH): Exploring the relationships between periventricular WMH, deep WMH, and total WMH burden. Stroke. 2005;36(1):50–55. 15576652 10.1161/01.STR.0000150668.58689.f2 PMC3816357

[43] Osburn HG . Coefficient alpha and related internal consistency reliability coefficients. Psychol Methods. 2000;5(3):343–355. 11004872 10.1037/1082-989x.5.3.343

[44] Baykara E, Gesierich B, Adam R, et al A novel imaging marker for small vessel disease based on skeletonization of white matter tracts and diffusion histograms. Ann Neurol. 2016;80(4):581–592. 27518166 10.1002/ana.24758

[45] van Veluw SJ, Hou SS, Calvo-Rodriguez M, et al Vasomotion as a driving force for paravascular clearance in the awake mouse brain. Neuron. 2020;105(3):549–561.e5. 31810839 10.1016/j.neuron.2019.10.033 PMC7028316

[46] Taoka T, Ito R, Nakamichi R, et al Diffusion-weighted image analysis along the perivascular space (DWI-ALPS) for evaluating interstitial fluid status: Age dependence in normal subjects. Jpn J Radiol. 2022;40(9):894–902. 35474438 10.1007/s11604-022-01275-0 PMC9441421

[47] Hsiao WC, Chang HI, Hsu SW, et al Association of cognition and brain reserve in aging and glymphatic function using diffusion tensor image-along the perivascular space (DTI-ALPS). Neuroscience. 2023;524:11–20. 37030632 10.1016/j.neuroscience.2023.04.004

[48] Charidimou A, Boulouis G, Gurol ME, et al Emerging concepts in sporadic cerebral amyloid angiopathy. Brain. 2017;140(7):1829–1850. 28334869 10.1093/brain/awx047 PMC6059159

[49] Xu J, Su Y, Fu J, et al Glymphatic dysfunction correlates with severity of small vessel disease and cognitive impairment in cerebral amyloid angiopathy. Eur J Neurol. 2022;29(10):2895–2904. 35712978 10.1111/ene.15450

[50] Peters N, Holtmannspotter M, Opherk C, et al Brain volume changes in CADASIL: A serial MRI study in pure subcortical ischemic vascular disease. Neurology. 2006;66(10):1517–1522. 16717211 10.1212/01.wnl.0000216271.96364.50

[51] Jolly AA, Nannoni S, Edwards H, Morris RG, Markus HS. Prevalence and predictors of vascular cognitive impairment in patients with CADASIL. Neurology. 2022;99(5):e453–e461. 35606149 10.1212/WNL.0000000000200607 PMC9421594

[52] Taniguchi A, Shindo A, Tabei KI, et al Imaging characteristics for predicting cognitive impairment in patients with cerebral autosomal dominant arteriopathy with subcortical infarcts and leukoencephalopathy. Front Aging Neurosci. 2022;14:876437. 35754959 10.3389/fnagi.2022.876437 PMC9226637

[53] Viswanathan A, Gschwendtner A, Guichard JP, et al Lacunar lesions are independently associated with disability and cognitive impairment in CADASIL. Neurology. 2007;69(2):172–179. 17620550 10.1212/01.wnl.0000265221.05610.70

