# Supplementary material for: Impaired cerebral interstitial fluid dynamics in cerebral autosomal dominant arteriopathy with subcortical infarcts and leucoencephalopathy
Source: Brain Commun. 2023 Dec 19;6(1):fcad349. doi: 10.1093/braincomms/fcad349 (PMC10757449; doi:10.1093/braincomms/fcad349)
Supplement: fcad349_Supplementary_Data [file fcad349_supplementary_data.pdf]

Supplementary Table 1. MRI parameters of the subjects with *NOTCH3* variants and healthy controls

Variables[a]	Group I: Healthy controls (*n* = 21)	Group II: Preclinical carriers (*n* = 37)	Group III: CADASIL patients (*n* = 44)	Age-, sex- adjusted *P* value, ANCOVA		
				II vs. I	III vs. I	II vs. III
Men	10 (47.6)	10 (27)	24 (59.1)	-	-	-
Age at exam, years	57.1 ± 12.9 (29~78)	49.5 ± 13.1 (27~77)	63.6 ± 9.1 (41~80)	-	-	-
DTI-ALPS index	1.57 ± 0.18 (1.26~1.92)	1.75 ± 0.29 (1.09~2.27)	1.36 ± 0.19 (1.07~1.77)	0.129	0.001*	< 0.001*
Brain parenchymal fraction, %	85.3 ± 5.1 (75.0~94.9)	87.9 ± 2.9 (82.3~94.0)	80.0 ± 4.0 (72.6~88.2)	0.308	0.001*	< 0.001*
Total WMH volume, ml	1.50 ± 1.55 (0.29~7.60)	16.16 ± 22.3 (0.33~94.14)	62.55 ± 29.38 (10.31~117.24)	< 0.001*	< 0.001*	< 0.001*
Deep WMH volume, ml	0.61 ± 0.64 (0~2.69)	8.32 ± 13.06 (0.15~64.65)	24.57 ± 15.81 (3.7~74.90)	0.005*	< 0.001*	< 0.001*
Periventricular WMH volume, ml	0.89 ± 1.00 (0.17~4.91)	8.90 ± 11.93 (0~44.67)	38.97 ± 18.42 (5.92~80.40)	< 0.001*	< 0.001*	< 0.001*
PSMD, $10^{-4} mm^2/s$	2.34 ± 0.38 (1.77~3.11)	2.75 ± 0.84 (1.77~5.19)	6.23 ± 2.24 (2.61~12.83)	< 0.001*	< 0.001*	< 0.001*
Lacune numbers	0.1 ± 0.4 (0~2)	0.9 ± 2.5 (0~12)	15.6 ± 10.6 (1~39)	0.01*	< 0.001*	< 0.001*
Cerebral microbleed counts	0.1 ± 0.5 (0~2)	1.3 ± 4.0 (0~22)	22.7 ± 29.3 (0~114)	0.05*	< 0.001*	< 0.001*

[a]Values are presented as mean ± SD (range) or *n* (%).

*$P < 0.05$

ANCOVA = Analysis of Covariance; CADASIL = Cerebral autosomal dominant arteriopathy with subcortical infarcts and leukoencephalopathy; DTI-ALPS = diffusion tensor image analysis along the perivascular space; WMH = white matter hyperintensity; PSMD = peak width of skeletonized mean diffusivity

Supplementary Table 2. Factors related to decreased DTI-ALPS index in the subjects with *NOTCH3* variants

Variables[a]	β (95% CI), *P* value	All subjects with *NOTCH3* variants (*n* = 81)	Preclinical carriers (*n* = 37)	CADASIL patients (*n* = 44)
Age at exam, years		-0.016 (-0.200, -0.120), < 0.001*	-0.015 (-0.020, -0.009), < 0.001*	-0.006 (-0.012, 0.0004), 0.068
Sex		-0.128 (-0.264, 0.007), 0.063	-0.003 (-0.226, 0.220), 0.978	-0.002 (-0.119, 0.115), 0.974
Hypertension		-0.272 (-0.401, -0.134), < 0.001*	-0.280 (-0.553, -0.007), 0.045*	-0.047 (-0.162, 0.068), 0.414
Diabetes		-0.043 (-0.273, 0.188), 0.713	0.118 (0.317, 0.553), 0.586	0.023 (-0.145, 0.190), 0.787
Hyperlipidemia		-0.134 (-0.280, 0.012), 0.71	-0.017 (-0.257, 0.224), 0.89	-0.084 (-0.199, 0.031), 0.15
Smoking		-0.141 (-0.301, 0.018), 0.082	-0.050 (-0.318, 0.218), 0.706	-0.082 (-0.205, 0.041), 0.188
Alcohol Consumption		-0.023 (-0.185, 0.140), 0.782	0.057 (-0.195, 0.309), 0.649	0.001 (-0.128, 0.130), 0.991
NOTCH3 R544C mutation or not		0.039 (-0.155, 0.233), 0.689	0.002 (-0.317, 0.320), 0.992	-0.029 (-0.177, 0.120), 0.701

[a]Factors, including age, sex, hypertension, diabetes, hyperlipidemia, smoking, alcohol consumption and *NOTCH3* R544C mutation, were independent variables and DTI-ALPS index was a dependent variable.

*$P < 0.05$

CADASIL = Cerebral autosomal dominant arteriopathy with subcortical infarcts and leukoencephalopathy; DTI-ALPS = diffusion tensor image analysis along the perivascular space

Supplementary Table 3. The associations between DTI-ALPS index and image features in the subjects with *NOTCH3* variants

Variables[a] β (95% CI), *P* value[b]	All subjects with *NOTCH3* variants (*n* = 81)	Preclinical carriers (*n* = 37)	CADASIL patients (*n* = 44)
Brain parenchymal fraction, %	7.918 (4.277, 11.559), < 0.001*	2.833 (-1.022, 6.688), 0.144	9.370 (3.283, 15.456), 0.003*
Total WMH volume, ml	-54.717 (-82.344, -27.089), < 0.001*	-14.541 (-42.239, 13.156), 0.293	-53.089 (-103.922, -2.256), 0.041*
PSMD, $10^{-4}mm^2/s$	-4.232 (-6.114, -2.349), < 0.001*	-0.574 (-1.418, 0.271), 0.176	-5.62 (-9.282, -1.958), 0.004*
Lacune number	-19.839 (-28.714, -10.965), < 0.001*	-0.538 (-3.827, 2.752), 0.741	-25.188 (-41.847, -8.530), 0.004*
Cerebral microbleed counts	-34.724 (-55.897, -13.552), 0.002*	1.107 (-4.860, 7.073), 0.708	-67.356 (-109.171, -25.543), 0.002*

[a]DTI-ALPS index was an independent variable and imaging markers including brain parenchymal fraction, total WMH volume, PSMD, lacune number, cerebral microbleed counts were dependent variables.

[b]Adjusted for age, sex and hypertension in the multivariate regression analysis.

*$P < 0.05$

CADASIL = Cerebral autosomal dominant arteriopathy with subcortical infarcts and leukoencephalopathy; DTI-ALPS = diffusion tensor image analysis along the perivascular space; WMH = white matter hyperintensity; PSMD = peak width of skeletonized mean diffusivity

Supplementary Table 4. The accuracy for DTI-ALPS index and vascular risk factors to distinguish symptomatic CADASIL patients from preclinical carriers

Imaging markers	AUC	Cut-off value[b]	Sensitivity	Specificity	PPV	NPV
Combined model[a]	0.900	0.384	95.5%	75.7%	82.4%	93.3%
DTI-ALPS index	0.866	1.454	75.0%	86.5%	86.8%	74.4%
Age, y	0.810	57.500	77.3%	75.7%	79.1%	73.7%
Sex (male)	0.660	-	59.1%	73.0%	72.2%	60.0%
Hypertension	0.717	-	56.8%	86.5%	83.3%	62.7%

[a]Combined model: including DTI-ALPS index, age, sex and hypertension in the receiver operating characteristic (ROC) curves analysis.

[b]The optimal cut-off values derived from Youden's index

AUC = area under the curve; CADASIL = Cerebral autosomal dominant arteriopathy with subcortical infarcts and leukoencephalopathy; DTI-ALPS = diffusion tensor image analysis along the perivascular space; NPV = negative predictive value; PPV = positive predictive value

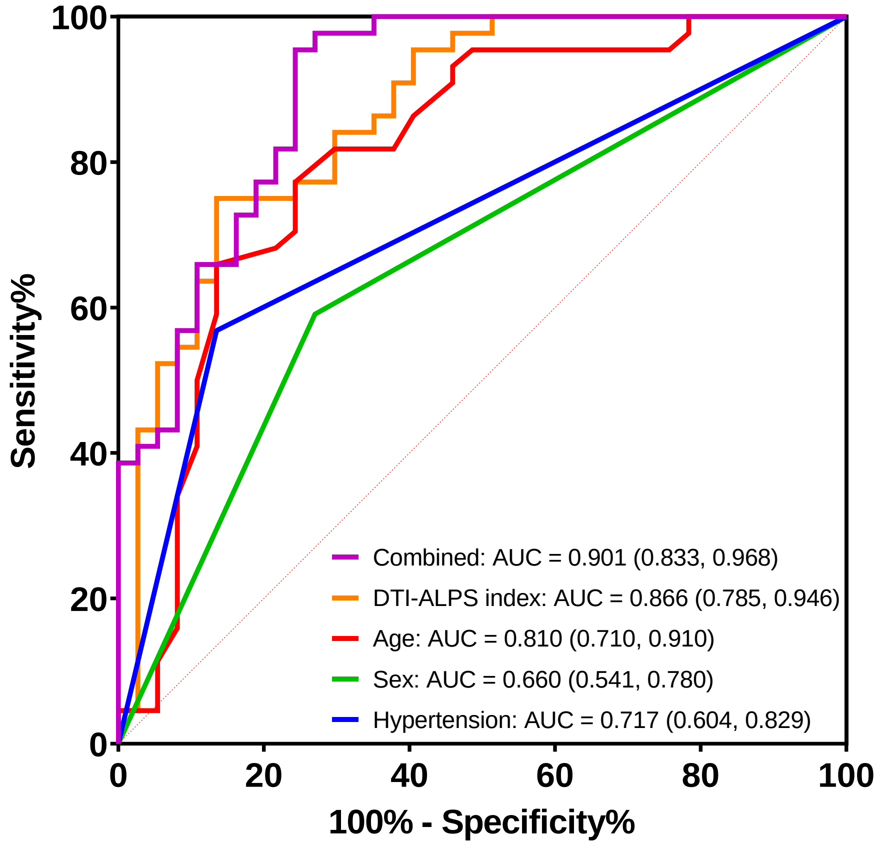

Supplementary Figure 1 The receiver operating characteristic (ROC) curve of single risk factors and the combined model for predicting *NOTCH3* variants carriers to develop clinical symptoms

AUC = area under curve; DTI-ALPS = diffusion tensor image analysis along the perivascular space; Combined = including age, sex, hypertension, and DTI-ALPS index in the ROC curve analysis
